# Supplementary material for: The Evolution and Characterization of the RNA Interference Pathways in Lophotrochozoa
Source: Genome Biol Evol. 2024 May 7;16(5):evae098. doi: 10.1093/gbe/evae098 (PMC11114477; doi:10.1093/gbe/evae098)
Supplement: evae098_Supplementary_Data [file evae098_supplementary_data.zip › Supplementary_materials.zip!/selected_miRNAs/NoGe_selected_miRNAs/known_miRNA_families_pdf_files/NMRB01003740.1_Notospermus_geniculatus_strain_Ushimado_scaffold3740,_whole_genome_shotgun_sequence_44401.pdf]

Provisional ID	:	NMRB01003740.1_Notospermus_geniculatus_strain_Ushimado_scaffold3740,_whole_genome_shotgun_sequence_44401
Score total	:	156820
Score for star read(s)	:	3.9
Score for read counts	:	156811.6
Score for mfe	:	1.4
Score for randfold	:	0
Score for cons. seed	:	3
Total read count	:	307591
Mature read count	:	306064
Loop read count	:	0
Star read count	:	1527

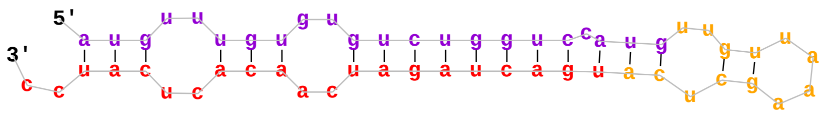

Genome coordinates: NMRB01003740.1 13268-13324

pre-miRNA sequence:
AUGUUUGUGUGUCUGGUCCAUGAUGUUUGUGUGUCUGGUCCAUGUUGUUAAAGCUCAUGACUAGAUCAACACUCAUCC

Mature sequence: UGACUAGAUCAACACUCAUCC

Aligned mature sequence of the *N. geniculatus* pre-miRNA with the top 5 hits from the BLAST search against the pre-miRNA mirGeneDB set:

```
                                  ********** **********
Nge_Mir-279                       ugacuagaucaacacucaucc-
Dpu-Mir-279-o6_3p                 ugacuagauccacacucaucca
Dma-Mir-279-o6_3p                 ugacuagauccacacucaucca
```
